# Epoxyeicosatrienoic Acid-Based Therapy Attenuates the Progression of Postischemic Heart Failure in Normotensive Sprague-Dawley but Not in Hypertensive *Ren-2* Transgenic Rats

**DOI:** 10.3389/fphar.2019.00159

**Published:** 2019-03-01

**Authors:** Jaroslav Hrdlička, Jan Neckář, František Papoušek, Zuzana Husková, Soňa Kikerlová, Zdenka Vaňourková, Zdenka Vernerová, Firat Akat, Jana Vašinová, Bruce D. Hammock, Sung Hee Hwang, John D. Imig, John R. Falck, Luděk Červenka, František Kolář

**Affiliations:** ^1^Institute of Physiology of the Czech Academy of Sciences, Prague, Czechia; ^2^Department of Physiology, Faculty of Science, Charles University, Prague, Czechia; ^3^Center for Experimental Medicine, Institute for Clinical and Experimental Medicine, Prague, Czechia; ^4^Department of Physiology, Faculty of Medicine, Ankara University, Ankara, Turkey; ^5^Department of Entomology and Nematology, UC Davis Comprehensive Cancer Center, University of California, Davis, Davis, CA, United States; ^6^Department of Pharmacology and Toxicology, Medical College of Wisconsin, Milwaukee, WI, United States; ^7^Department of Biochemistry, University of Texas Southwestern, Dallas, TX, United States

**Keywords:** epoxyeicosatrienoic acid, soluble epoxide hydrolase, chronic heart failure, hypertension, myocardial infarction, echocardiography

## Abstract

Epoxyeicosatrienoic acids (EETs) and their analogs have been identified as potent antihypertensive compounds with cardio- and renoprotective actions. Here, we examined the effect of EET-A, an orally active EET analog, and *c*-AUCB, an inhibitor of the EETs degrading enzyme soluble epoxide hydrolase, on the progression of post-myocardial infarction (MI) heart failure (HF) in normotensive Hannover Sprague-Dawley (HanSD) and in heterozygous *Ren-2* transgenic rats (TGR) with angiotensin II-dependent hypertension. Adult male rats (12 weeks old) were subjected to 60-min left anterior descending (LAD) coronary artery occlusion or sham (non-MI) operation. Animals were treated with EET-A and *c*-AUCB (10 and 1 mg/kg/day, respectively) in drinking water, given alone or combined for 5 weeks starting 24 h after MI induction. Left ventricle (LV) function and geometry were assessed by echocardiography before MI and during the progression of HF. At the end of the study, LV function was determined by catheterization and tissue samples were collected. Ischemic mortality due to the incidence of sustained ventricular fibrillation was significantly higher in TGR than in HanSD rats (35.4 and 17.7%, respectively). MI-induced HF markedly increased LV end-diastolic pressure (P_ed_) and reduced fractional shortening (FS) and the peak rate of pressure development [+(dP/dt)_max_] in untreated HanSD compared to sham (non-MI) group [P_ed_: 30.5 ± 3.3 vs. 9.7 ± 1.3 mmHg; FS: 11.1 ± 1.0 vs. 40.8 ± 0.5%; +(dP/dt)_max_: 3890 ± 291 vs. 5947 ± 309 mmHg/s]. EET-A and *c*-AUCB, given alone, tended to improve LV function parameters in HanSD rats. Their combination amplified the cardioprotective effect of single therapy and reached significant differences compared to untreated HanSD controls [P_ed_: 19.4 ± 2.2 mmHg; FS: 14.9 ± 1.0%; +(dP/dt)_max_: 5278 ± 255 mmHg/s]. In TGR, MI resulted in the impairment of LV function like HanSD rats. All treatments reduced the increased level of albuminuria in TGR compared to untreated MI group, but neither single nor combined EET-based therapy improved LV function. Our results indicate that EET-based therapy attenuates the progression of post-MI HF in HanSD, but not in TGR, even though they exhibited renoprotective action in TGR hypertensive rats.

## Introduction

Current experimental and clinical research into pathophysiological mechanisms of cardiovascular diseases provided series of evidences suggesting an increased activity of soluble epoxide hydrolase (sEH) in several heart and kidney disorders (Imig, 2012; Oni-Orisan et al., 2014; Campbell et al., 2017). sEH is an enzyme responsible for rapid conversion of cytochrome P450 arachidonic acid epoxygenase metabolites, the epoxyeicosatrienoic acids (EETs), to inactive or less active dihydroxyeicosatrienoic acids (DHETs). It has been shown that EETs have beneficial action to combat many cardiovascular diseases and their progression including hypertension, ischemic heart diseases, chronic heart failure (CHF), diabetes mellitus, chronic kidney diseases etc. (reviewed in Qiu et al., 2011; Jamieson et al., 2017; Imig, 2018). Therefore, sEH inhibitors represent a potential class of drugs for treating various cardiovascular diseases.

Despite major improvements in the therapy of cardiac disorders, the prevalence of CHF is rising (Braunwald, 2013). In most instances, CHF is the irreversible and final consequence of heart injury associated with high morbidity and mortality. The progression of left ventricle (LV) dysfunction following acute myocardial infarction (MI) is a predominant cause of CHF (Roger, 2013). Regarding the therapeutic effects of EETs on post-MI remodeling, it has been shown that chronic treatment of normotensive rats or mice with sEH inhibitors reduce the progression of LV systolic dysfunction (Li et al., 2009; Merabet et al., 2012; Kompa et al., 2013; Sirish et al., 2013).

It is well known that specific metabolic pathways, including sEH, quickly terminate the biological activity of EETs (Spector and Norris, 2007). To circumvent this limitation of endogenous EETs, several synthetic and more stable EET analogs with markedly longer half-life and promising cardioprotective actions have been developed (Campbell et al., 2017). Previously, it has been shown that NUDSA, the first generation EET analog, increased LV function and decreased cardiac fibrosis in mice after MI (Cao et al., 2015).

Altogether, the EET therapy based on EET analogs or sEH inhibitors can limit a harmful myocardial remodeling associated with the progression of post-MI CHF, however, their combined action was never analyzed. Therefore, here we examined the effect of EET-A (a third generation of EET analog with better water solubility and improved oral bioavailability) and *c*-AUCB (sEH inhibitor), given alone or combined, on the progression of post-MI CHF in normotensive Hannover Sprague-Dawley (HanSD) and in heterozygous *Ren-2* transgenic rats (TGR) with angiotensin II (Ang II)-dependent form of hypertension. Previously, it has been shown that *c*-AUCB reduced mortality in TGR subjected to aorto-caval fistula, a well-defined model of CHF due to volume overload (Červenka et al., 2015a). Moreover, the same EET-based therapy (EET-A and *c*-AUCB) administered before MI effectively reduced high blood pressure and decreased the incidence of life-threatening ventricular fibrillation in hypertensive TGR (Červenka et al., 2018).

## Materials and Methods

### Animals and Experimental Protocol

HanSD rats and TGR were bred at the Center of Experimental Medicine of the Institute for Clinical and Experimental Medicine in Prague and housed in a controlled environment (23°C, 12 h light-dark cycle; light from 6:00 AM) with free access to water and standard chow diet. The study was conducted in accordance with the Guide for the Care and Use of Laboratory Animals published by the National Academy of Science, National Academy Press, Washington, DC. The experimental protocols were approved by the Animal Care and Use Committee of the Institute of Physiology of the Czech Academy of Sciences.

Adult male HanSD rats and TGR (12 weeks old; *n* = 76 and 90, respectively) were subjected to 60-min regional ischemia or sham operation as described earlier (Hrdlička et al., 2016). Briefly, animals were anesthetized with sodium pentobarbital (60 mg/kg, i.p., Sigma-Aldrich, United States). Intubated rats were ventilated (Ugo Basile, Italy) with room air at 68 strokes/min (tidal volume of 1.2 ml/100 g body weight) and their rectal temperature was maintained between 36.5 and 37.5°C with a heating pad. A left thoracotomy was performed and the pericardium was removed to reveal the location of the left anterior descending (LAD) coronary artery. Then a silk ligature 6/0 (Chirmax, Czech Republic) was placed around the LAD coronary artery about 1–2 mm distal to the origin and an occlusive snare was placed around it. The ends of the suture were threaded through a polyethylene tube. After the surgical preparation, the rats were allowed to stabilize for 10 min before the ischemic intervention. Myocardial ischemia was induced by tightening the ligature around the coronary artery. Sham (non-MI) surgery was performed identically without occlusion. Characteristic changes in myocardial color and the incidence of ischemic arrhythmias verified the complete coronary artery occlusion. At the start of reperfusion, the snare was released, the chest was closed, air was removed from the thorax and spontaneously breathing animals recovering from anesthesia were housed in separate cages and received analgesia (Ibuprofen, 6 mg/day p.o.) for 3 consecutive days.

Twenty-four hours after surgery, rats of both strains were randomly assigned based on their treatment to the following groups: (i) untreated Sham-operated (non-MI), (ii) post-MI untreated controls, and rats treated by (iii) EET-A (10 mg/kg/day, p.o.), (iv) *c*-AUCB (1 mg/kg/day, p.o.), and (v) a combination of EET-A and *c*-AUCB (10 mg/kg/day and 1 mg/kg/day, respectively, p.o.).

### Echocardiography

LV geometry and function were assessed by echocardiography 3 days prior to MI and 5 weeks post-MI using GE Vivid 7 Dimension (GE Vingmed Ultrasound, Horten, Norway) with a 12 MHz linear matrix probe M12L (Hrdlička et al., 2016). Animals were anesthetized with 2% isoflurane (Forane, Abbott Laboratories, Queenborough, United Kingdom) mixed with room air, placed on a heating pad and their rectal temperature was maintained between 36.5 and 37.5°C. Basic 2-D and M-mode in both long and short axis and 4-D mitral flow and pulmonary artery (PA) pulse Doppler measurements were recorded. Heart rate (HR) and following parameters of LV geometry were assessed: end-diastolic and systolic LV cavity diameter (LVDd, LVDs), LV cavity length (LVLd, LVLs), LV cavity area in short axis (LVAd, LVAs), anterior wall thickness (AWTd, AWTs), and posterior wall thickness (PWTd, PWTs). Fractional shortening (FS) and relative wall thickness (RWT) were derived as follows: FS = 100^∗^[(LVDd-LVDs)/LVDd]; RWT = 100^∗^[(AWTd+PWTd)/LVDd].

### Heart Catheterization

At the end of the study, rats anesthetized with 2% isoflurane were subjected to LV catheterization through the right carotid artery using the SPR-407 microtip pressure catheter as described previously (Lee et al., 2008) and data were acquired using MPVS 300 (Millar, Houston, TX, United States), PowerLab 8/30 (ADInstruments, Oxford, United Kingdom). End-diastolic, systolic, and developed pressure and peak rate of pressure development and decline (+(dP/dt)max, -(dP/dt)max, respectively) were assessed from 5 consecutive pressure cycles using LabChart Pro (ADInstruments, Oxford, United Kingdom).

### Plasma Monocyte Chemoattractant Protein-1 (MCP-1)

After catheterization, blood was collected from the right ventricle, centrifuged and plasma samples were frozen in liquid nitrogen and stored in −80°C. Plasma concentration of MCP-1 was measured by a quantitative sandwich enzyme immunoassay technique, using a commercially available ELISA kit (BMS631INST, Invitrogen by Thermo Fischer Scientific, Austria).

### Kidney Injury Markers

Twenty-four hour urine samples were collected at the end of the five-week post-MI follow-up period. Albumin and cystatin C were measured by a quantitative sandwich enzyme immunoassay technique, using a commercially available ELISA kits (ERA3201-1, AssayPro, MO, United States; EK1109, BOSTER Biological Technology Co., Ltd., CA, United States). Urine creatinine was determined using Liquick Cor-Creatinine kit without deproteinization (PZ Cormay S.A., Poland). In alkaline solution, picrate reacts with creatinine to form a yellow-red 2,4,6-trinitrocyclohexadienate. The color intensity measured by a photometer at 500 nm is proportional to the creatinine concentration. Albumin data were normalized to the creatinine data. Sodium and potassium levels in urine samples were measured using flame photometer BWB-XP (BWB Technologies, Great Britain).

Kidneys were immersion-fixed in 10% neutral buffered formalin and paraffin embedded. Tissue sections were cut into 4 μm slices for use in histology protocols. Tissue slices were de-paraffinized, re-hydrated and stained with hematoxylin/eosin and periodic acid-Schiff reaction and examined using Nikon Eclipse Ni-E. Slides were evaluated in a blind fashion. Renal damage was expressed as a total index, the sum of glomerulosclerosis and cortical tubulointerstitial injury (CTI).

Glomerulosclerosis/hyalinosis (GSI) was defined as segmental solidification of glomerular capillary tuft, segmental collapse, obliteration of capillary lumen and accumulation of hyaline. One hundred glomeruli per section were randomly selected and evaluated using semiquantitative scoring method. Degree of sclerosis in each glomerulus was graded subjectively: grade 0, normal; grade 1, sclerotic area up to 25% (minimal); grade 2, sclerotic area 25–50% (moderate); grade 3, sclerotic area 50–75% (moderate-severe); and grade 4, sclerotic area 75–100% (severe). GSI index was calculated using the following formula: GSI = [(1^∗^n1)+(2^∗^n2)+(3^∗^n3)+(4^∗^n4)]/n0+n1+n2+n3+n4, where n is the number of glomeruli in each grade of glomerulosclerosis.

Cortical tubulointerstitial injury (CTI) was defined as inflammatory cell infiltration, tubular dilatation and/or atrophy, or interstitial fibrosis. Lesions were assessed for at least 30 random and non-overlapping fields in a cortex and were graded semiquantitatively using the following scale: 0, no abnormal findings; 1, mild (up to 25% of the cortex); 2, moderate (25–50% of the cortex); 3, severe (more than 50% of cortex). CTI index was calculated using the following formula: CTI = [(1^∗^n1)+(2^∗^2n)+(3^∗^3n)]/30 (fields), where nx is the number of fields in each grade.

### Statistical Analysis

Data are expressed as mean ± SEM. Statistical evaluation were done using GraphPad Prism 6 (GraphPad Software, San Diego, CA, United States). The incidence of mortality was evaluated by Fisher’s exact test. For multiple comparison of between group differences one-way analysis of variance (ANOVA) and Holm-Sidak’s multiple comparison *post hoc* test were used. The values exceeding 95% probability limits (*P* < 0.05) were considered statistically significant.

## Results

### Weight Parameters, Mortality, and Plasma MCP-1 Level

**FIGURE 1:**
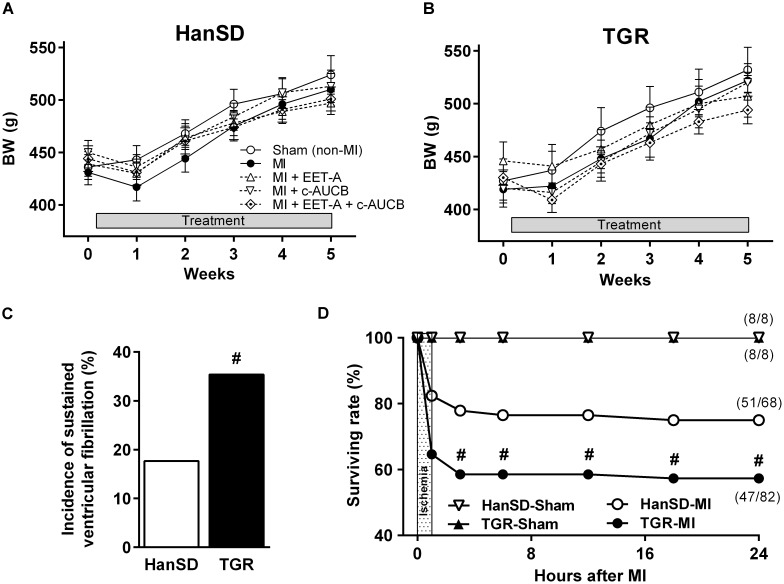
Body weight of Hannover Sprague-Dawley (HanSD; **A**) and *Ren-2* transgenic rats (TGR; **B**) before myocardial infarction (MI) and during 5 weeks of post-MI period and in Sham (non-MI) operated animals. Rats were treated with epoxyeicosatrienoic acid analog (EET-A) or soluble epoxide hydrolase inhibitor (*c*-AUCB), given alone or combined. The incidence of sustained ventricular fibrillation during ischemia **(C)** and 24 h survival rate **(D)** in HanSD and TGR subjected to MI. Values are means ± SEM. **^#^***P* < 0.05 vs. HanSD.

At the beginning of the study, the experimental groups did not differ in body weight (BW). A slight reduction in BW was observed one week after MI. From week two post-MI, BW gain occurred and by the end of the study BW did not differ among the groups (Figure 1A,B).

In HanSD rats, the acute ischemic mortality was 17.7% due to the incidence of sustained ventricular fibrillation (sVF); mortality increased to 25.0% by 24 h after MI (Figure 1C,D). Only two additional untreated HanSD rats died during 5 weeks of post-MI period. In TGR, the acute and 24 h mortality were significantly higher (35.4 and 42.7%, respectively) compared to HanSD rats (Figure 1C,D). Moreover, three untreated rats and four rats from each therapy group of TGR died during post-MI period; the post-MI mortality did not differ among TGR groups. Overall, 72.1% (49 out of 68) of HanSD rats and 39.0% (32 out of 82) of TGR subjected to MI survived till the end of the study.

As summarized in Table 1, in untreated HanSD rats MI led to the significant increase in the relative heart weight (HW/BW) compared to Sham (non-MI) group (3.23 ± 0.14 mg/g vs. 2.71 ± 0.14 mg/g). Sham (non-MI) TGR exhibited higher HW/BW (3.22 ± 0.11 mg/g) than HanSD rats. As compared to HanSD rats, MI had no significant effect on the relative heart weight in TGR (Table 1). Neither single nor combined EET-based therapy significantly affected HW/BW in both strains compared to corresponding untreated MI groups.

**Table 1:** Relative weights of lung, heart and right kidney of Hannover Sprague-Dawley (HanSD) and *Ren-2* transgenic rats (TGR) subjected to sham operation (non-MI) or myocardial infarction (MI) and treated with epoxyeicosatrienoic acid analog (EET-A) or soluble epoxide hydrolase inhibitor (*c*-AUCB), given alone or combined, for 5 weeks since 24 h after MI.

	n	Heart/BW (mg/g)	Lungs/BW (mg/g)	Kidney/BW (mg/g)
**HanSD**				
Sham (non-MI)	8	2.71 ± 0.14	2.94 ± 0.07	3.20 ± 0.07
MI	11	3.23 ± 0.14^†^	7.04 ± 0.82^†^	3.17 ± 0.15
MI + EET-A	14	3.03 ± 0.09	4.84 ± 0.64	3.43 ± 0.06
MI + *c*-AUCB	10	3.10 ± 0.10	6.63 ± 1.28	3.44 ± 0.09
MI + EET-A + *c*-AUCB	14	3.00 ± 0.05	4.78 ± 0.75	3.46 ± 0.04
**TGR**				
Sham (non-MI)	8	3.22 ± 0.11	3.05 ± 0.13	3.57 ± 0.10
MI	8	3.44 ± 0.11	6.82 ± 0.59^†^	3.11 ± 0.09^†^
MI + EET-A	8	3.19 ± 0.08	6.46 ± 0.97	3.19 ± 0.04
MI + *c*-AUCB	10	3.19 ± 0.14	6.93 ± 0.74	3.14 ± 0.09
MI + EET-A + *c*-AUCB	6	3.11 ± 0.11	7.55 ± 1.29	3.07 ± 0.04

*BW, body weight; n, number of animals. Values are means ± SEM. ^†^P < 0.05 MI vs. corresponding Sham (non-MI) group.*

The progression of post-MI CHF was associated with significant increase in lungs weight by 139 and 123%, respectively, in untreated HanSD rats and TGR compared to corresponding non-MI controls. In HanSD rats (but not in TGR) treated with EET-A alone or combined with *c*-AUCB, relative lung weight increased only by 65 and 63%, respectively (Table 1). Nevertheless, the observed decreases did not reach statistical significance compared to untreated HanSD rats.

The concentration of MCP-1, a chemotactic cytokine, was analyzed as a marker of systemic inflammation in plasma. As shown in Supplementary Figure S1, neither MI nor EET-based therapy significantly affected MCP-1 levels in both strains at the end of study.

### Kidney Injury Markers

**FIGURE 2:**
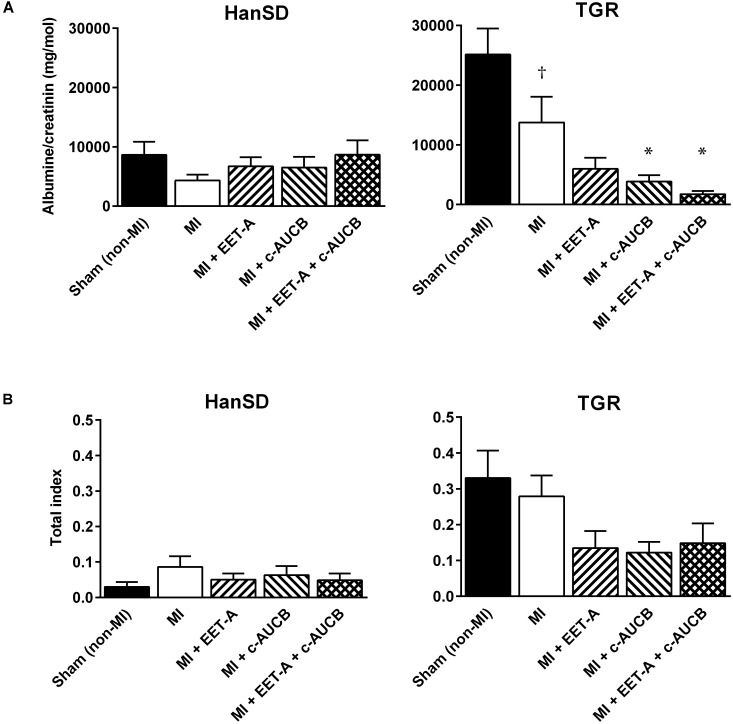
Albumine/Creatinine clearance **(A)** and the total index of kidney injury (the sum of glomerulosclerosis and cortical tubulointerstitial injury; **B**) in Hannover Sprague-Dawley (HanSD) and *Ren-2* transgenic rats (TGR) subjected to sham operation (non-MI) or myocardial infarction (MI) and treated with epoxyeicosatrienoic acid analog (EET-A) or soluble epoxide hydrolase inhibitor (*c*-AUCB), given alone or combined for 5 weeks since 24 h after MI. Values are means ± SEM; **^†^***P* < 0.05 MI vs. corresponding Sham (non-MI) group; ^∗^*P* < 0.05 vs. corresponding MI group.

Neither MI nor EET-based therapy affected kidney weight, albuminuria, and kidney total injury score in HanSD rats (Table 1 and Figure 2). In untreated TGR, increased levels of kidney injury markers were observed. MI slightly decreased the relative kidney weight, albuminuria and kidney total injury score (by 13, 45, and 27%). EET-based therapy had no additional effects on kidney weight. EET-A decreased albuminuria in TGR by 56%, though the effect was not significant. However, *c*-AUCB and the combined treatment reduced albuminuria significantly (by 72 and 87%, respectively). EET-based therapy decreased the total index of kidney injury by 43–57% in TGR (Figure 2B) but the differences did not reach statistical significances. Finally, neither MI nor EET-based therapy significantly affected urinary excretion of sodium and potassium as well as cystatin C, a marker of renal tubular dysfunction, at the end of study (Supplementary Figure S2).

### Heart Geometry and Function Assessed by Echocardiography

**FIGURE 3:**
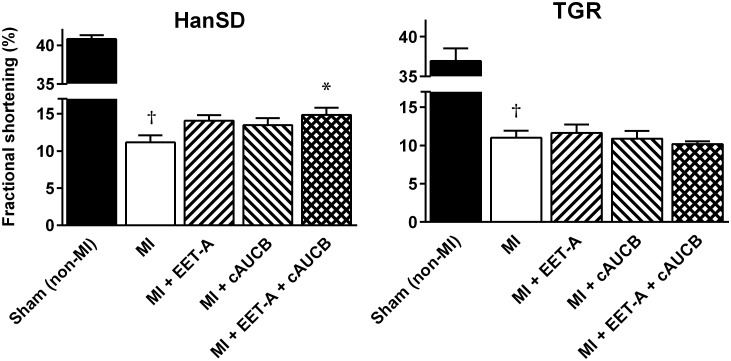
Fractional shortening in Hannover Sprague-Dawley (HanSD) and *Ren-2* transgenic rats (TGR) subjected to Sham (non-MI) operation or myocardial infarction (MI) and treated with epoxyeicosatrienoic acid analog (EET-A) or soluble epoxide hydrolase inhibitor (*c*-AUCB), given alone or combined for 5 weeks since 24 h after MI. Values are means ± SEM; **^†^***P* < 0.05 MI vs. corresponding Sham (non-MI) group; ^∗^*P* < 0.05 vs. corresponding MI group.

At the beginning of the study (before MI), AWTd and PWTd reached 2.10 ± 0.07 mm and 2.09 ± 0.04 mm, respectively, in Sham (non-MI) HanSD rats; LV systolic function, determined as FS, was 42.1 ± 0.9%. In Sham (non-MI) TGR, concentric LV hypertrophy and systolic dysfunction was observed (AWTd: 2.60 ± 0.09 mm; PWTd: 2.61 ± 0.07 mm; FS: 36.9 ± 1.6%). Similar differences in diastolic wall thickness and systolic function between Sham (non-MI) HanSD rats and TGR were also observed at the end of study (Table 2 and Figure 3).

**Table 2:** Echocardiographic parameters of left ventricle (LV) and heart rate (HR) in Hannover Sprague-Dawley (HanSD) and *Ren-2* transgenic rats (TGR) subjected to sham operation (non-MI) and myocardial infarction (MI) and treated with epoxyeicosatrienoic acid analog (EET-A) or soluble epoxide hydrolase inhibitor (*c*-AUCB), given alone or combined for 5 weeks since 24 h after MI.

	AWTd (mm)	LVDd (mm)	PWTd (mm)	AWTs (mm)	LVDs (mm)	PWTs (mm)	RWT (%)	HR (bpm)
**HanSD**								
Sham (non-MI)	2.21 ± 0.09	8.82 ± 0.20	2.28 ± 0.09	3.44 ± 0.11	5.27 ± 0.16	3.39 ± 0.10	51.1 ± 2.1	360 ± 14
MI	1.69 ± 0.08^†^	11.70 ± 0.17^†^	2.41 ± 0.10	1.66 ± 0.07^†^	10.37 ± 0.21^†^	2.88 ± 0.13^†^	35.1 ± 0.9^†^	327 ± 11
MI + EET-A	1.61 ± 0.05	11.24 ± 0.17	2.26 ± 0.07	1.62 ± 0.06	9.65 ± 0.17	2.92 ± 0.09	34.6 ± 0.7	347 ± 8
MI + *c*-AUCB	1.61 ± 0.05	11.13 ± 0.16	2.34 ± 0.10	1.62 ± 0.05	9.62 ± 0.20	2.95 ± 0.11	35.6 ± 1.2	355 ± 7
MI + EET-A + *c*-AUCB	1.60 ± 0.07	11.13 ± 0.24	2.16 ± 0.05	1.70 ± 0.12	9.50 ± 0.29^*^	2.83 ± 0.07	34.0 ± 1.1	356 ± 7
**TGR**								
Sham (non-MI)	2.64 ± 0.10	8.52 ± 0.17	2.57 ± 0.10	3.66 ± 0.14	5.44 ± 0.13	3.42 ± 0.10	65.1 ± 2.8	373 ± 13
MI	1.95 ± 0.08^†^	11.65 ± 0.29^†^	2.41 ± 0.09	2.03 ± 0.15^†^	10.35 ± 0.33^†^	3.16 ± 0.11	35.0 ± 1.6^†^	353 ± 11
MI + EET-A	1.88 ± 0.08	11.19 ± 0.30	2.41 ± 0.05	1.93 ± 0.13	9.90 ± 0.35	3.17 ± 0.06	38.1 ± 2.2	351 ± 15
MI + *c*-AUCB	1.84 ± 0.06	11.54 ± 0.17	2.40 ± 0.09	1.96 ± 0.14	10.28 ± 0.24	3.00 ± 0.09	35.3 ± 1.0	343 ± 10
MI + EET-A + *c*-AUCB	1.85 ± 0.08	11.47 ± 0.16	2.43 ± 0.11	1.94 ± 0.18	10.30 ± 0.16	3.10 ± 0.14	35.4 ± 1.7	321 ± 16

*AWTd, diastolic anterior wall thickness; LVDd, diastolic LV diameter; PWTd, diastolic posterior wall thickness; AWTs, systolic anterior wall thickness; LVDs, systolic LV diameter; PWTs, systolic posterior wall thickness; RWT, relative wall thickness; bpm, beats per minute. Values are means ± SEM; **^†^**P < 0.05 MI vs. corresponding Sham (non-MI) group; ^∗^P < 0.05 vs. corresponding MI group.*

As summarized in Table 2, MI without treatment resulted in significant decreases in diastolic and systolic AWT and increased LVD in both strains compared to corresponding Sham (non-MI) groups. In the untreated MI group of HanSD rats, systolic PWT was also significantly decreased. These changes in LV geometry were reflected in significantly decreased RWT in both strains (by 31% in HanSD rats and 48% in TGR; Table 2). In HanSD rats (but not in TGR), EET-A or *c*-AUCB, given alone, decreased both diastolic (11.24 ± 0.17 and 11.13 ± 0.16 mm, respectively) and systolic (9.65 ± 0.17 and 9.62 ± 0.20 mm, respectively) LVD compared with untreated MI controls (LVDd: 11.70 ± 0.17; LVDs: 10.37 ± 0.21) but the differences did not reach statistical significances. The combined treatment decreased LVDs significantly (9.50 ± 0.29 mm) compared to untreated MI group. Other parameters of LV geometry were not significantly affected by the treatments in any strain (Table 2).

MI markedly decreased LV systolic function expressed as FS in untreated animals of both strains (to 11.1 ± 1.0% and 11.0 ± 0.9%, respectively; Figure 3). In HanSD rats, EET-A and *c*-AUCB, given alone, improved FS to 14.1 ± 0.8% and 13.5 ± 0.9%, respectively, but the increase was not statistically significant. The combined administration of EET-A and *c*-AUCB amplified the cardioprotective effect of single therapy and significantly improved FS to 14.9 ± 1.0% compared with untreated HanSD controls. In TGR, neither single nor combined EET-based therapy affected LV systolic function (Figure 3).

Progression of post-MI heart failure was associated with changes of mitral flow time parameters. In both untreated HanSD rats and TGR, MI significantly increased the LV filling peak velocity, prolonged the isovolumic contraction time, and shortened the filling time, but had no effect on the ejection time and isovolumic relaxation time (Table 3). In HanSD rats, *c*-AUCB alone significantly reduced the prolongation of isovolumic contraction time. Combined EET-based therapy shortened the isovolumic contraction time and also prolonged the filling time compared to MI untreated controls. Neither single nor combined EET-based therapy affected CHF-associated changes in time parameters of mitral flow in TGR.

**Table 3:** Doppler echocardiography of mitral and pulmonary artery flow in Hannover Sprague-Dawley (HanSD) and *Ren-2* transgenic rats (TGR) subjected to sham operation (non-MI) and myocardial infarction (MI) and treated with epoxyeicosatrienoic acid analog (EET-A) or soluble epoxide hydrolase inhibitor (*c*-AUCB), given alone or combined for 5 weeks since 24 h after MI.

	Mitral flow					Pulmonary artery flow			
	Vm_max_ (m.s^−1^)	FT (ms)	IVCT (ms)	ET (ms)	IVRT (ms)	Vpa_max_ (m.s^−1^)	Vpa_mean_ (m.s^−1^)	AT (ms)	ETpa (ms)
**HanSD**									
Sham (non-MI)	1.11 ± 0.04	64.1 ± 3.6	13.9 ± 1.2	67.9 ± 4.4	23.7 ± 1.8	1.06 ± 0.05	0.46 ± 0.02	27.5 ± 1.5	95.1 ± 3.0
MI	1.32 ± 0.04^†^	45.2 ± 1.7^†^	49.2 ± 5.3^†^	61.5 ± 2.0	29.5 ± 1.8	0.77 ± 0.05^†^	0.31 ± 0.02^†^	25.4 ± 1.6	92.1 ± 2.2
MI + EET-A	1.29 ± 0.04	49.6 ± 1.6	40.0 ± 5.5	63.7 ± 1.8	25.5 ± 2.2	0.92 ± 0.04	0.39 ± 0.02^*^	28.0 ± 1.4	91.1 ± 1.2
MI + *c*-AUCB	1.31 ± 0.06	46.6 ± 1.8	30.5 ± 3.2^*^	59.9 ± 3.5	30.6 ± 2.7	0.89 ± 0.04	0.37 ± 0.02	27.5 ± 1.9	89.5 ± 2.4
MI + EET-A + *c*-AUCB	1.17 ± 0.05	54.0 ± 2.0^*^	31.4 ± 3.7^*^	57.9 ± 1.9	26.3 ± 1.4	0.92 ± 0.04	0.39 ± 0.02^*^	27.1 ± 1.4	90.7 ± 1.3
**TGR**									
Sham (non-MI)	1.20 ± 0.04	59.5 ± 3.0	14.6 ± 1.6	63.8 ± 2.0	21.4 ± 2.6	1.08 ± 0.03	0.47 ± 0.02	28.2 ± 1.3	89.8 ± 1.7
MI	1.51 ± 0.06^†^	44.8 ± 1.9^†^	35.9 ± 4.7^†^	59.1 ± 3.8	32.0 ± 4.3	0.80 ± 0.06^†^	0.32 ± 0.03^†^	25.2 ± 1.4	88.1 ± 1.5
MI + EET-A	1.36 ± 0.06	47.0 ± 1.3	41.3 ± 7.3	61.2 ± 4.8	31.3 ± 7.2	0.89 ± 0.05	0.37 ± 0.03	25.4 ± 0.7	87.7 ± 1.5
MI + *c*-AUCB	1.38 ± 0.06	47.0 ± 2.5	46.1 ± 5.8	64.8 ± 2.9	20.7 ± 1.7	0.83 ± 0.04	0.34 ± 0.03	26.3 ± 1.3	90.4 ± 2.0
MI + EET-A + *c*-AUCB	1.27 ± 0.09	43.1 ± 3.5	56.2 ± 9.4	61.0 ± 4.7	29.0 ± 3.8	0.75 ± 0.04	0.30 ± 0.03	26.7 ± 1.7	91.2 ± 1.3

*Vm_max_, left ventricle filling peak velocity; FT, filling time; IVCT, isovolumic contraction time; ET, left ventricle ejection time; IVRT, isovolumic relaxation time; Vpa_max_, peak velocity; Vpa_mean_, mean velocity; AT, acceleration time; ETpa, pulmonary artery ejection time. Values are means ± SEM; ^†^P < 0.05 MI vs. corresponding Sham (non-MI) group; ^∗^P < 0.05 vs. corresponding MI group.*

MI reduced the PA peak and mean velocities, but did not change the PA ejection time and acceleration time in both untreated post-MI groups (Table 3). In HanSD rats, the PA mean velocity significantly increased after *c*-AUCB as well as combined treatments. Neither single nor combined EET-based therapy affected CHF-associated changes in PA flow in TGR.

### Heart Function and Blood Pressure Assessed by Catheterization

**FIGURE 4:**
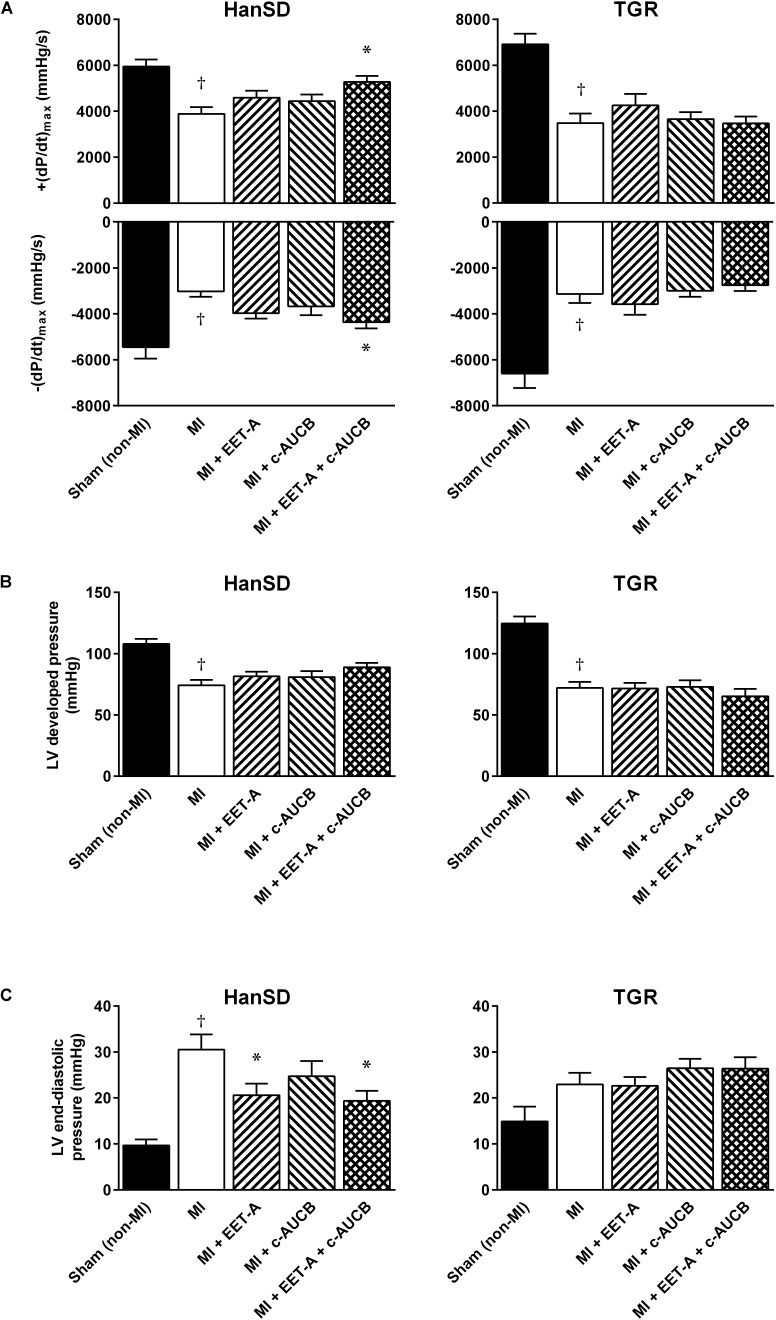
Left ventricle peak rates of pressure development and fall **(A)**, developed pressure **(B)**, and end-diastolic pressure **(C)** in Hannover Sprague-Dawley (HanSD) and *Ren-2* transgenic rats (TGR) subjected to Sham (non-MI) operation or myocardial infarction (MI) and treated with epoxyeicosatrienoic acid analog (EET-A) or soluble epoxide hydrolase inhibitor (*c*-AUCB), given alone or combined for 5 weeks since 24 h after MI. Values are means ± SEM; **^†^***P* < 0.05 MI vs. corresponding Sham (non-MI) group; ^∗^*P* < 0.05 vs. corresponding MI group.

As demonstrated in Figure 4, the progression of post-MI CHF resulted in impaired LV contractile function. In MI untreated HanSD and TGR groups, the peak rate of pressure development [+(dP/dt)_max_] markedly decreased to 3890 ± 291 and 3485 ± 417 mmHg/s, respectively, compared to corresponding Sham (non-MI) groups (5947 ± 301 and 6910 ± 462 mmHg/s, respectively, Figure 4A). In HanSD rats, EET-A or *c*-AUCB, given alone, improved +(dP/dt)_max_ to 4596 ± 297 and 4442 ± 287 mmHg/s, respectively, though the effect was not significant. The combined treatment provided the stronger cardioprotective effect (5278 ± 255 mmHg/s) than single therapies reaching significant difference compared to untreated HanSD controls; the peak value of pressure decline [-(dP/dt)_max_] exhibited similar changes (Figure 4A).

In untreated HanSD and TGR, MI induced significant systolic blood pressure reduction to 104.7 ± 2.6 and 97.0 ± 6.4 mmHg, respectively, compared to corresponding Sham (non-MI) animals (117.7 ± 3.7 and 139.6 ± 4.4 mmHg, respectively). Neither single nor combined treatment affected systolic blood pressure which varied between 102 and 108 mmHg in HanSD rats and 93–99 mmHg in TGR. In both strains, EET-based therapy did not significantly affect LV developed pressure in animals subjected to MI (Figure 4B). However, EET-A treatment, given alone or in combination, significantly reduced high LV end-diastolic pressure to 20.6 ± 2.5 and 19.4 ± 2.2 mmHg, respectively, compared with 30.5 ± 3.3 mmHg in untreated HanSD rats (Figure 4C). In TGR, neither single nor combined EET-based therapy affected post-MI LV dysfunction (Figure 4).

## Discussion

The main finding of the study is that the therapeutic administration of combined EET-based therapy after MI slowed down the progression of post-MI CHF in HanSD rats. As compared to normotensive strain, neither single nor combined treatment by EET analog and sEH inhibitor affected the progression of post-MI CHF in transgenic rats with Ang II-dependent hypertension. These effects were demonstrated by echocardiography as well as the direct LV catheterization.

In the present study, TGR were used as a well characterized experimental model of monogenetic hypertension of renal origin. The TGR strain [TGR(mRen2)27] was created by Mullins et al. (1990) as a rat model with an additional expression of the murine renin. Heterozygous TGR males reach maximum blood pressure at the age of 8–9 weeks (Lee et al., 1996). It has been demonstrated that TGR develop pathophysiological changes of the heart such as LV hypertrophy and myocardial fibrosis (Bachmann et al., 1992). With respect to the heart function, TGR exhibited unchanged (Habibi et al., 2011; Ma et al., 2012; Neckář et al., 2012; Červenka et al., 2015a; Kovács et al., 2016) or slightly lower LV systolic function (Whaley-Connell et al., 2007; De Mello et al., 2013), depending on measured heart parameters and used methods. Further, it has been shown that TGR have increased mortality compared to normotensive HanSD rats when subjected to volume overload (Červenka et al., 2015a,b; Kala et al., 2018). Finally, there is a single paper dealing with the progression of post-MI CHF in TGR (Connelly et al., 2013). Using TGR females, it demonstrated the LV function impairment after MI comparable to our present study. It also showed that combined pharmacological treatment with angiotensin converting enzyme and direct renin inhibition blunted the progression of post-MI CHF.

The present study follows on from our recent report (Červenka et al., 2018) which analyzed the preventive effect of the same EET-based therapy on the acute cardiac ischemic tolerance in HanSD rats and TGR. We demonstrated that EET-A and *c*-AUCB, given alone or combined before MI (two-week treatment), did not affect the infarct size in both strains and had no additional effects on hearts of HanSD rats. However, both single and combined EET-based therapy lowered high blood pressure, decreased LV hypertrophy and reduced the increased incidence of ischemia-induced ventricular fibrillation in hypertensive TGR (Červenka et al., 2018). These findings provided the impetus to conduct the current experimental study to determine effects of EET-A and *c*-AUCB, given alone or combined after MI.

Here we examined effects of single and combined EET-A treatment on the progression of post-MI CHF. The third generation of orally active EET agonist analogs, including EET-A, demonstrated great potential for therapy of cardiovascular and kidney diseases in rat and mouse models. Indeed, the most promising compounds (EET-A and EET-B) were validated as powerful 14,15-EET analogs (Falck et al., 2014; Khan et al., 2014; Campbell et al., 2017). It has been shown that these novel and orally active EET analogs provided heart and kidney protection comparable with that of native EETs (Skibba et al., 2017; Neckář et al., 2018). Previously, Imig’s group demonstrated that EET-A markedly reduced cisplatin-induced nephrotoxicity and mitigated radiation nephropathy in rats (Khan et al., 2013; Hye Khan et al., 2016). EET-A also ameliorated the deleterious effects of high fat diet-induced metabolic abnormalities in obese mice (Singh et al., 2016). In the same mouse model (db/db mice), EET-A treatment improved LV systolic function (Cao et al., 2017). Recently, we have shown that the continuous treatment by another 14,15-EET analog EET-B before and after MI reduced post-MI mortality and the progression of LV systolic dysfunction in spontaneously hypertensive rats (Neckář et al., unpublished) Compared to predominant EET analogs-mediated protection against end-organ injury, their antihypertensive action is rather inconsistent; rodent models with various genetic background of hypertension differ in their sensitivity to EET-based therapy. Hence, EET-A reduced blood pressure in various forms of Ang II-dependent models of hypertension in rats (Neckář et al., 2012; Hye Khan et al., 2014; Červenka et al., 2018) and in mice with high fat diet-induced obesity (Singh et al., 2016). On the other hand, EET-A or EET-B did not exhibit any antihypertensive action in Dahl salt-sensitive rats, Goldblatt hypertensive rats, Cyp1a1-Ren-2 transgenic rats, and in spontaneously hypertensive rats (Hye Khan et al., 2013; Alánová et al., 2015; Jíchová et al., 2016; Neckář et al., unpublished). Therefore, it seems that the protective action of EET analogs against end-organ injury is independent of blood pressure reduction in hypertensive animal models.

The progression of post-MI CHF in the present study was associated with decreased albuminuria and the total index of kidney injury in untreated TGR compared to Sham (non-MI) controls. We speculate that these findings can reflect blood pressure reduction after MI. Indeed, it is well known that post-MI CHF decreases blood pressure in hypertensive rats due to insufficient myocardial function (Nishikimi et al., 1995; Mori et al., 1998; Wiemer et al., 2001) which can result in reduced kidney injury. Further, EET-based therapy almost eliminated albuminuria and decreased kidney injury score in TGR. This finding is not surprising because sEH inhibitors and EET analogs, respectively, represent promising and powerful therapies to prevent the progression of various chronic kidney diseases to renal failure (Imig, 2015; Fan and Roman, 2017). Similarly, preventive treatment of TGR by the same EET-based therapy before MI resulted in kidney protection (Červenka et al., 2018). The absence of any differences in other urinary markers of renal injury suggests that post-MI progression of CHF did not substantially damage kidney in both strains.

Previously, it has been demonstrated that acute exogenous administration of EETs or inhibition of sEH attenuated the increase of endothelial cell permeability and lung injury after acute ischemia/reperfusion or lipopolysaccharides administration (Townsley et al., 2010; Chen et al., 2015; Tao et al., 2016). Further, long-lasting sEH inhibitor treatment reduced bleomycin-induced pulmonary injury (Dong et al., 2017). It seems that EETs can protect against various lung diseases associated with inflammation and oxidative stress like asthma and chronic obstructive pulmonary disease (Yang et al., 2015, 2017). In line with these observations, EET-based therapy moderately limited CHF-induced lungs edema and improved PA flow in HanSD rats in the present study.

It has been shown that chronic treatment with sEH inhibitors reduced the progression of post-MI LV systolic dysfunction in normotensive animals (Li et al., 2009; Merabet et al., 2012; Kompa et al., 2013; Sirish et al., 2013). The beneficial effect of different sEH inhibitors was associated with increased EETs levels (Li et al., 2009; Sirish et al., 2013). Similarly, *c*-AUCB (given alone or in combination with EET-A) increased the myocardial concentration of endogenous EETs in both HanSD rats and TGR (Červenka et al., 2018). Moreover, Merabet et al. (2012) demonstrated that the cardioprotective action of sEH treatment was abolished by co-administration with an inhibitor of cytochrome P450 epoxygenase, the enzyme producing EETs from arachidonic acid. Therefore, EETs can play a role in the progression of post-MI CHF in normotensive rats, which is in line with our present study.

The effect of sEH inhibitors on the progression of CHF associated etiologies other than ischemic heart disease is inconsistent. Indeed, it has been reported that other sEH inhibitors such as AEPU, AUDA and TPPU reduced the development of cardiac hypertrophy and diminished adverse cardiac remodeling in normotensive mice subjected to pressure overload due to thoracic aortic constriction (Xu et al., 2006; Sirish et al., 2013). On the other hand, another sEH inhibitor GSK2256294 did not reverse LV dysfunction induced by pressure overload in both mice and rats, in spite of the fact that the increased an EETs-to-DHETs ratio was observed (Morgan et al., 2013). Similarly, *c*-AUCB did not alter LV contractility in hypertensive TGR and Fawn-hooded rats as well as normotensive HanSD and Fawn-hooded low-pressure rats subjected to volume overload (Červenka et al., 2015a,b; Vacková et al., 2019).

The overall biology of both mimics of epoxy fatty acids (EpFA) and sEH inhibitors are anticipated to be similar and in some cases additive. An intrinsic problem with sEH inhibitors is that they can only preserve the EpFA that are present. This has the advantage of making it difficult to over dose, but a disadvantage is that the sEH inhibitors cannot correct for abnormally low levels of EpFA. The mimics on the other hand do not require endogenous production of EpFA for their biological activity. However, the mimics only mimic a single isomer of the complex array of EpFAs present in an organism while sEH inhibitors to some degree preserve all EpFA. The preservation is likely to be related to the endogenous ratios of endogenous EpFA. Thus each pharmaceutical approach offers endogenous advantages and limitations. One can anticipate situations where the effect of the two drug classes would likely be additive. As individual EpFA mimics and sEH inhibitors are selected for development each will present unique pharmacokinetic parameters which will offer specific limitations and assets (Shen and Hammock, 2012).

## Conclusion

In conclusion, our results showed that combined EET-based therapy reduced the progression of post-MI CHF in normotensive HanSD rats. Even though they exhibited renoprotective action, neither single nor combined treatment by EET-A and *c*-AUCB affected the extent of post-MI CHF in *Ren-2* transgenic rats with Ang II-dependent form of hypertension. Cardioprotective efficacy of EET-based therapy against the progression of CHF varies depending on experiment model and protocol, and associated comorbidities.

## Data Availability

All datasets generated for this study are included in the manuscript and/or the Supplementary Files.

## Author Contributions

JN primarily conceived and designed the study, performed experiments, and wrote the manuscript. JH performed experiments, analyzed and interpreted the data, and wrote the manuscript. BH, SH, JI, and JF designed and synthesized *c*-AUCB and EET-A. FP, ZH, SK, ZVa, ZVe, FA, JV, LČ, and FK were involved in performing the experiments, data collection, analysis and interpretation, contributed to the intellectual content and editing of the manuscript, and approved its final version.

## Conflict of Interest Statement

The authors declare that the research was conducted in the absence of any commercial or financial relationships that could be construed as a potential conflict of interest.

## Supplementary Material

FIGURE S1 Plasma concentration of monocyte chemoattractant protein-1 (MCP-1) at the end of study in Hannover Sprague-Dawley (HanSD) and *Ren-2* transgenic rats (TGR) subjected to Sham (non-MI) operation or myocardial infarction (MI) and treated with epoxyeicosatrienoic acid analog (EET-A) or soluble epoxide hydrolase inhibitor (*c*-AUCB), given alone or combined for 5 weeks since 24 h after MI. Values are means ± SEM.

FIGURE S2 Urinary sodium **(A)**, potassium **(B)** and cystatin c **(C)** excretion at the end of study in Hannover Sprague-Dawley (HanSD) and *Ren-2* transgenic rats (TGR) subjected to Sham (non-MI) operation or myocardial infarction (MI) and treated with epoxyeicosatrienoic acid analog (EET-A) or soluble epoxide hydrolase inhibitor (*c*-AUCB), given alone or combined for 5 weeks since 24 h after MI. Values are means ± SEM.
